# A glutamate concentration‐biased allosteric modulator potentiates NMDA‐induced ion influx in neurons

**DOI:** 10.1002/prp2.859

**Published:** 2021-09-03

**Authors:** Blaise M. Costa, Lina Cortes, Brittney Mehrkens, Douglas N. Bledsoe, Bryanna N. Vacca, Tullia V. Johnston, Rehan Razzaq, Dhanasekaran Manickam, Bradley G. Klein

**Affiliations:** ^1^ Center for One Health Research Virginia Maryland College of Veterinary Medicine Virginia Tech Blacksburg Virginia USA; ^2^ Edward Via Virginia College of Osteopathic Medicine (VCOM Blacksburg Virginia USA; ^3^ School of Neuroscience Virginia Tech Blacksburg Virginia USA; ^4^ Syngene International Bangalore India; ^5^ Present address: Virginia Commonwealth University Richmond Virginia USA; ^6^ Present address: University of North Carolina Chapel Hill North Carolina USA

**Keywords:** anti‐NMDA receptor encephalopathy, biased allosteric modulator (BAM), NMDA receptor, schizophrenia

## Abstract

Precisely controlled synaptic glutamate concentration is essential for the normal function of the N‐methyl D‐aspartate (NMDA) receptors. Atypical fluctuations in synaptic glutamate homeostasis lead to aberrant NMDA receptor activity that results in the pathogenesis of neurological and psychiatric disorders. Therefore, glutamate concentration‐dependent NMDA receptor modulators would be clinically useful agents with fewer on‐target adverse effects. In the present study, we have characterized a novel compound (CNS4) that potentiates NMDA receptor currents based on glutamate concentration. This compound alters glutamate potency and exhibits no voltage‐dependent effect. Patch‐clamp electrophysiology recordings confirmed agonist concentration‐dependent changes in maximum inducible currents. Dynamic Ca^2+^ and Na^+^ imaging assays using rat brain cortical, striatal and cerebellar neurons revealed CNS4 potentiated ion influx through native NMDA receptor activity. Overall, CNS4 is novel in chemical structure, mechanism of action and agonist concentration‐biased allosteric modulatory effect. This compound or its future analogs will serve as useful candidates to develop drug‐like compounds for the treatment of treatment‐resistant schizophrenia and major depression disorders associated with hypoglutamatergic neurotransmission.

## Graphical abstract

Novel NMDA receptor modulator selectively potentiates whole‐cell current response based on glutamate concentration.

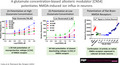

**Abbreviations**
Ca^2+^
: calcium ions
cDNA: complementary DNA
E18: embryonic day‐18
GluN: glutamate receptor NMDA subtype
HEK293T: human embryonic kidney 293 transfectable
MTS: 3‐(4,5‐dimethylthiazol‐2‐yl)‐5‐(3‐carboxymethoxyphenyl)‐2‐(4‐sulfophenyl)‐2H‐tetrazolium
Na^+^
: sodium ions
NMDA: N‐methyl D‐aspartate
TEVC: two electrode voltage clamp

## 1 INTRODUCTION

N‐methyl D‐aspartate (NMDA) is an analog of the excitatory neurotransmitter L‐glutamic acid first synthesized by Jeff Watkins in the early 1960s.^1^, ^2^ NMDA, with the concurrent binding of the co‐agonist D‐serine or glycine, selectively activates a specific population of ionotropic glutamate receptors, known as NMDA receptors (NMDARs) that non‐selectively conduct monovalent and divalent ions across brain cells at depolarizing membrane potential which unbinds the otherwise‐blocking Mg^2+^ ions. Functional NMDARs are composed of two identical glycine‐binding GluN1 subunits and two identical or different glutamate‐binding GluN2 subunits, of which there are four subtypes GluN2A‐D. Two glycine‐binding GluN3 (A‐B) subunits can also assemble with GluN1 to form excitatory glycine receptors.^3^, ^4^ Each non‐GluN1 subunit confers distinct spatiotemporal expression and biophysical properties that result in varying agonist affinity, magnesium sensitivity, ion conductance, activation kinetics, open probability, mean open time, cellular localization and downstream signaling mechanism.^5^ Recent studies demonstrate that activation of extrasynaptic NMDARs signal pro‐apoptotic^6^, ^7^ events whereas synaptic NMDAR activation promotes pro‐survival mechanisms.^8^, ^9^, ^10^ GluN2A subunits are reported to be expressed in the synapse and GluN2B and 2D at the extrasynaptic site.^8^, ^9^ Recent studies identified synaptic expression of GluN2B & 2D containing NMDARs receptors in the hippocampal interneurons.^11^ containing This heterogeneity enables NMDARs to carry out the complicated tasks as required for normal glutamatergic neurotransmission.^12^

Pulsatile release and subsequent changes in glutamate concentration in the synapse are essential for maintaining normal brain physiology.^13^, ^14^, ^15^, ^16^, ^17^, ^18^ Glutamate concentration exceeds 1 mM in the synaptic cleft following an action potential for less than 10 ms and rapidly returns to less than 20 nM between two consecutive release events due to high‐affinity glutamate uptake by neurons and glial cells.^17^, ^18^ Furthermore, glutamate spillover from the synapse, that occurs during the rapid rise and fall event, causes a glutamate concentration gradient across the penumbra of the synapse, commonly known as the extrasynaptic site. This concentration gradient at extrasynaptic sites varies over three orders of magnitude, ranging from 0.02 to 30 µM as identified by electrophysiology and microdialysis experiments.^19^, ^20^

NMDAR hypofunction is the second leading cause for the pathogenesis of psychiatric disorders; the first being the well‐studied role of dopamine (e.g., the dopamine hypothesis).^21^, ^22^, ^23^, ^24^ There is genetic, biochemical and pharmacologic evidence supporting the contribution of NMDAR hypofunction to the pathophysiology of schizophrenia,^21^ and the comorbidity of substance abuse.^25^ Furthermore, a mutation in the GRIN2B gene (E413G), which is known to cause ~50‐fold reduction in glutamate potency for GluN2B subunit, has been associated with developmental abnormalities.^26^, ^27^ These findings reveal that point mutation‐induced submaximal activation of NMDARs may result in clinically significant and incurable symptoms of neurological and psychiatric disorders. Therefore, selectively potentiating hypo‐activated NMDAR function would be an appropriate strategy to treat the symptoms of schizophrenia and other related conditions that are not improved by the currently available medications.^21^, ^28^, ^29^, ^30^ Furthermore, drugs that can recognize conformational changes, which occur due to varying glutamate concentrations, might produce less unwanted effects through the intended target.

In this study, through ongoing computational and experimental screening efforts,^31^, ^32^, ^33^, ^34^, ^35^ we have identified a compound from the NIH PubChem database (CID# 3794169) that contains 111 million unique chemical structures. Previously known biological effects of this compound can be obtained from PubChem. This compound was chosen based on its chemical similarity with previously known NMDA receptor potentiators. This compound was synthesized and pharmacologically studied for its glutamate concentration‐dependent effect on different NMDAR subtypes.

## 2 MATERIALS AND METHODS

### 2.1 Synthesis of 4‐fluoro‐N‐(2‐(pyridin‐3‐yl)piperidine‐1‐carbonothioyl)benzamide

**FIGURE 1:**
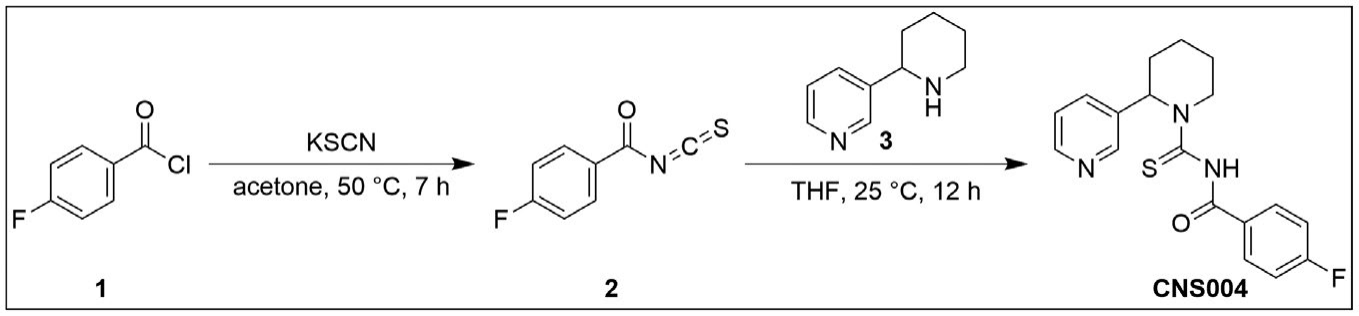
Synthetic route and chemical structure of CNS004. Anabasine (piperidinylpyridine alkaloid) based new thiourea was synthesized in two steps using thiocarbamoylation reaction. The starting 4‐fluorobenzoyl isothiocyanate was synthesized in situ by heating 4‐fluorobenzoyl chloride 1 with potassium thiocyanate in acetone. Further reaction of fluorobenzoyl isothiocyanate 2 with anabasine 3 in tetrahydrofuran (THF) at room temperature yielded 4‐fluoro‐N‐(2‐(pyridin‐3‐yl)piperidine‐1‐carbonothioyl)benzamide (CNS004). This compound is referred as CNS4 in the text. More details on CNS4 synthesis provided in the methods section. Detailed synthetic route and experimental procedures provided in the Supporting Information Figure 1‐1

Anabasine, piperidinylpyridine alkaloid‐based new thiourea was synthesized in two steps using thiocarbamoylation reaction. The starting 4‐fluorobenzoyl isothiocyanate was synthesized in situ by heating 4‐fluorobenzoyl chloride 1 with potassium thiocyanate in acetone. Further reaction of fluorobenzoyl isothiocyanate 2 with anabasine 3 in tetrahydrofuran (THF) at room temperature yielded 4‐fluoro‐N‐(2‐(pyridin‐3‐yl)piperidine‐1‐carbonothioyl)benzamide (CNS4). The synthesized compound was confirmed by 1H‐NMR & LCMS analysis and HPLC purity >99%. Detailed synthetic route and experimental procedures are provided in the Supporting Information data Figure 1‐1. This compound can be obtained from Blaise Costa through reasonable request.

### 2.2 Two electrode voltage clamp (TEVC) electrophysiology in xenopus oocytes

NMDAR constructs: cDNA encoding the NMDAR1a subunit (GluN1a) was obtained from Dr. Nakanishi. cDNA encoding the GluN2B (pci_sepGluN2B) was developed initially in the Malinow lab^36^ and purchased from Addgene (cat# 23998). cDNA encoding the GluN2C and GluN2D were purchased from GenScript. Mutated GluN1, 2A and 2B cDNA constructs capable of assembling as GluN1/2A/2B triheteromeric (1/2AB) receptors^37^ were obtained from Dr. Paoletti (Laboratoire de Neurobiologie, CNRS). These constructs have been previously tested for 1/2AB receptor activity of GluN1/2A receptor‐selective potentiators.^38^ Plasmids were linearized with NotI (GluN1a) or Avrll (GluN2B), or BstB1 (GluN2C and GluN2D) and transcribed in vitro with T7 (GluN1a, GluN1/2A, GluN2B, GluN2C, & GluN2D) RNA polymerase using the mMessage mMachine transcription kits (Invitrogen by Thermo Fisher Scientific).

### 2.3 GluN subunit expression and electrophysiology in Xenopus oocytes

Stage IV frog oocytes were obtained from Xenopus‐I. NMDAR subunit cRNAs were suspended in nuclease‐free sterile water. GluN1A, GluN2B, GluN2C, GluN2D, and GluN1/2A/2B cRNAs were mixed in a ratio of 1:1–3. 50 nl of the final cRNA mixture was microinjected (40–70 ng total) into the oocyte cytoplasm. Oocytes were incubated in ND‐96 solution at 18^◦^C prior to electrophysiological recordings (1–3 days).

### 2.4 Dose–response curves

Electrophysiological responses were measured using a standard two‐microelectrode voltage‐clamp workstation [Warner Instruments (Hamden, Connecticut) model OC‐725C] designed to provide a fast clamp of large cells. The recording buffer contained 116 mM NaCl, 2 mM KCl, 0.3 mM BaCl_2_, and 5 mM HEPES, pH 7.4. Response magnitude was determined in xenopus oocytes expressing different NMDAR subunits (GluN1/2A, 1/2B, 1/2C, 1/2D, and 1/2A/2B) by the steady plateau response elicited by bath application of different agonist concentrations. 0.3 or 100 or 300 µM L‐glutamate and 100 μM glycine were used to activate the receptors at a holding potential of −60 mV. Response amplitudes for functional NMDARs were generally between 0.1 and 2 μA. After obtaining a steady‐state response to agonist application, agonist plus CNS4 in different concentrations were applied (1–100 μM of CNS4 compound), using an 8‐channel perfusion system (Automate Scientific), on the oocytes and the responses were digitized for quantification (Digidata 1550A and pClamp‐10, Molecular Devices). Dose–response relationships were fit to an appropriate curve‐fitting equation using GraphPad Prism‐7. Nonlinear regression was used to calculate EC_50_ and/or percentage maximal response. Curve fittings were done using the following equations as performed in the previous studies^34^, ^39^: *Y* = 100/(1 + 10^(LogEC50‐X)^) or *Y* = Bottom + (Top − Bottom)/(1+10^(LogEC50‐X)^). Statistical significance was determined at the overall alpha level .05, using appropriate statistical methods as described in each figure captions.

### 2.5 Current–voltage (I–V) relationship experiments

I–V relationship was studied using xenopus oocytes expressing different NMDAR subunits (GluN1/2A, 1/2B, 1/2C, and 1/2D) using 100 μM L‐glutamate +100 μM glycine application at different holding potentials starting from −90 mV up to +30 mV in 10 mV intervals. After obtaining a steady‐state response to different agonist applications, agonist plus 100 μM CNS4 or agonist plus 40 μM MgCl_2_ plus 100 μM CNS4 was applied. Forty micrometers MgCl_2_ was chosen from previously published experiments,^32^ since this was the Mg^2+^ IC_50_ (at −60 mV) for GluN1/2A receptors. Data points were aligned by least‐square fit by third‐order polynomial equation (*Y* = B0 + B1*X +B2*X ^2^ + B3*X ^3^), except for GluN1/2B that needed a fourth‐order polynomial equation.

### 2.6 HEK‐293T cells & whole‐cell patch‐clamp electrophysiology

Whole‐cell patch‐clamp electrophysiology studies were carried out in the HEK‐293 cells, expressing recombinant NMDARs that lack native functional NMDARs.^40^, ^41^, ^42^ Equal quantity of (1 µg) cDNA for GluN1a, GluN2 (A or B or C or D) subunits were co‐transfected 24–48 h before patch‐clamp electrophysiology assay. Activation of NMDAR by ambient glutamate from the cell culture media was inhibited (to avoid excitotoxicity) by adding 50 µM memantine into the culture media during transfection.^43^ Cells were carefully washed before performing experiments and were used for the electrophysiology experiments after 24–48 h incubation at 37°C with 5%CO_2_. The whole‐cell patch‐clamp electrophysiology assay was performed using semi‐automated patch‐clamp equipment, Port‐a‐Patch (Nanion Technologies GmbH). Following are the constituents of various solutions used for patch‐clamp electrophysiology: internal solution [(mM) NaCl 10, EGTA 20, CsF 110, HEPES 10], Mg‐free external (recording) solution [(mM) NaCl 140, KCl 4, CaCl_2_ 2, HEPES 10, D‐GlucoseMonohydrate 5], and Mg‐free seal enhancer solution [(mM) NaCl 80, KCl 3, CaCl_2_ 35, HEPES 10]. Nanion NPC chips with 2–3.5 mOhms resistance were used for the HEK‐293 cell recordings. Agonist concentrations used for this set of experiments are provided in the Figure 5.

### 2.7 Primary rat brain neuron culture

All animal experimental procedures and housing have been approved by the Institutional Animal Care and Use Committee (IACUC) protocol #18‐015 of Virginia Tech. Embryonic day 18 (E18) rat brain tissue samples were obtained from adult pregnant SD rats. Rat brain primary cortical, striatal, and cerebellar neurons were cultured on poly‐d‐lysine coated 96‐well plates for 14 days in vitro (DIV14) before using for the experiments. Primary rat neuron culture was performed as previously published.^44^ Briefly, each well of a 96 well plate was loaded with 50 000 cells and grown in 200 µl neurobasal media supplemented with B27, glutamax, penicillin, streptomycin. One hundred microliters of media was replaced with fresh media once in 4 days. Eight to thirteen fetus were obtained from each pregnant rat. Brain tissue from these fetus were pooled to obtain the neurons. Cortical, striatal, and cerebellar neuron assays were carried out at different time points.

Dynamic calcium assay was carried out, using Fluo‐8 no‐wash kit (abcam, ab112129), as per the manufacturer's instructions with minor modifications to fit with experimental necessities. On DIV14, all 200 μl of media was removed and replaced it with 100 μl of HBSS and 100 μl of Fluo‐8 dye loading solution. The plates were incubated at 37°C for 30 min and at room temperature for 30 min. A volume of 100 µL of test chemicals (3× of desired final concentration) was added to the plates immediately before running the calcium flux assay and read the fluorescence intensity at 490/525 nm (ex/em) using Synergy microplate reader, BioTek, VT. Costar 96‐well clear bottom black side plates were read from the bottom, nine times, with 60 s intervals between each read. Temperature was set at 37°C and reading speed was 100 ms.

For Na^+^ assay, DIV14 neurons were washed with HBSS and treated with freshly prepared CoroNa green‐AM dye (ThermoFisher, cat # C36676) and incubated for 45 min in 37°C. Plates were washed twice with HBSS before adding drug solutions dissolved in HBSS, and read at 492/516 nm (ex/em) using a plate reader. RFU values of background control and treatments were plotted to identify the effect of CNS4 on NMDA‐induced Na^+^ flux in the neurons.

## 3 RESULTS

### 3.1 Glutamate concentration‐dependent effect of CNS4 on NMDAR subtypes

**FIGURE 2:**
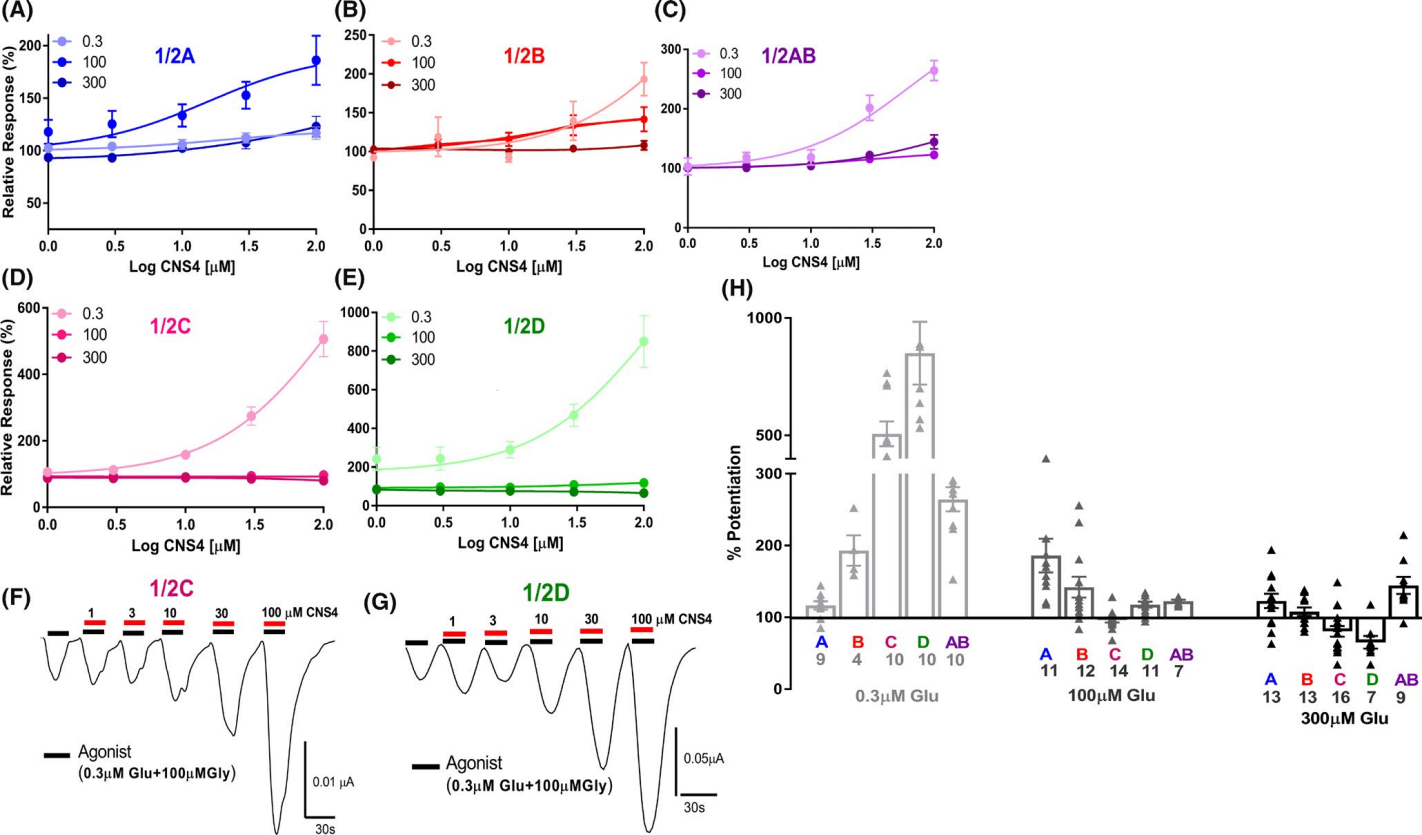
Glutamate concentration‐dependent effect of CNS4 on GluN1/2A, 1/2B, 1/2C, 1/2D, and 1/2A and 1/2B subunit containing triheteromeric (1/2AB) receptors. (A–E) CNS4 dose–response studies using three (0.3, 100 & 300 µM) different agonist concentrations as presented by gradient colors in each data set. All agonist solutions contained 100 µM glycine. (F–G) Representative traces of GluN1/2C and 1/2D receptor potentiation with 0.3 µM glutamate. (H) Histograms summarize the effect of 100 µM CNS4 dose in each agonist concentrations studied. Receptor subtypes are labelled as A(blue), B(red), C(maroon), D(green), & AB(purple) below respective histograms. Numbers underneath each alphabet represent the number of recordings made for each subtype. Number of oocytes used for each concentration: 0.3 µM Glu (1/2A, 8; 1/2B, 8; 1/2C, 6; 1/2D, 7), 100 µM Glu (1/2A, 4; 1/2B, 4; 1/2C, 4; 1/2D, 4), 300 µM Glu (1/2A, 4; 1/2B, 3; 1/2C, 6; 1/2D, 4). Y‐axis 100% denotes agonist‐induced maximal activation. Numbers more than hundred represent the percentage potentiation. Numbers less than hundred represent percentage inhibition in the presence of 100 µM CNS4. Results confirming the intermediate inhibitory effect of ifenprodil on GluN1/2AB receptors is provided in the Supporting Information data figure 2‐1

The synthetic route and chemical structure of CNS4 has been provided in Figure 1. CNS4 potentiates agonist‐induced NMDAR currents in a glutamate concentration‐dependent manner (Figure 2A–H). Results obtained from TEVC electrophysiology assay reveal that CNS4 potentiates 0.3 µM glutamate evoked whole‐cell recombinant GluN1/2C currents [506.34% ± 52.9% (average ± SEM, agonist alone induced maximum current is normalized to 100%)] and 1/2D currents (850.35% ± 133.9%), Figure 2D,E,H. Interestingly, CNS4 had almost no effect (<20% of potentiation) on GluN1/2C & 1/2D receptors when a higher concentration (100 & 300 µM) of glutamate was used to activate the receptor. A pair of CNS4 dose–response traces show the potentiation of 0.3 µM glutamate current responses in GluN1/2C and 1/2D receptors (Figure 2F,G), respectively. GluN1/2A receptor currents were better potentiated (186.08% ± 23.5%) by 100 µM glutamate than 300 (122.87% ± 9.9%) or 0.3 µM (1116.61% ± 5.9%) glutamate. When activated with 0.3 µM glutamate, CNS4 potentiated (193.22% ± 21.2%) GluN1/2B and had a negligible effect on GluN1/2A (Figure 2H). Since GluN2A and GluN2B subunits are canonical representatives of NMDARs, the combination of these subunits containing tri‐heteromeric GluN1/2AB receptors also have been studied. Recent studies identified GluN1/2AB receptors as predominant NMDAR subtypes expressed throughout the hippocampus and cortex.^45^, ^46^, ^47^, ^48^, ^49^, ^50^ In the 1/2AB receptors, similar to the activity on GluN1/2B, CNS4 potentiated (264.37% ± 16.9%) 0.3 µM glutamate‐induced currents and minimally affected 100 µM (122.65% ± 1.9%) and 300 µM (144.47% ± 11.8%) glutamate evoked currents. To confirm the expression of GluN1/2AB receptors and their pharmacological effect, we have performed a control experiment with a known GluN1/2B selective compound, ifenprodil. Since GluN1/2AB receptors contain both 2A and 2B subunits, it was hypothesized that ifenprodil should give an intermediate EC_50_ on the GluN1/2AB receptor compared to 1/2A and 1/2B receptors. IC_50_ values (1/2A:202.06 ± 25.09 µM; 1/2B: 4.66 ± 1.33 µM; 1/2AB, 20.30 ± 9.05 µM) obtained from the ifenprodil dose–response curves provided in the Supporting Information data Figure 2‐1.

### 3.2 CNS4 alters glutamate potency based on the composition of GluN2 subunits

**FIGURE 3:**
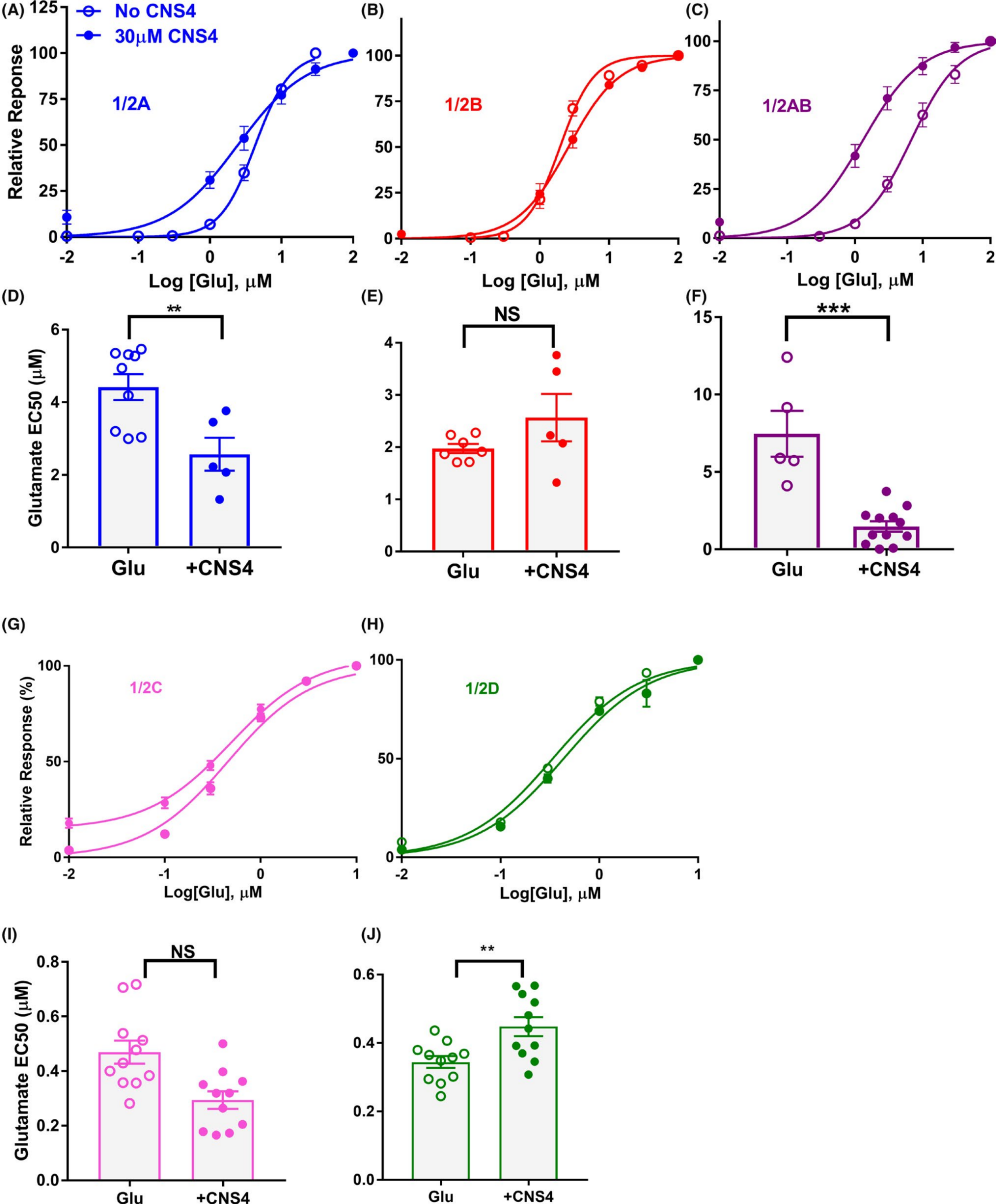
CNS4 alters agonist potency in NMDA receptor subtypes. Glutamate dose–response curve in the absence (open circles) or presence (filled circles) of 30uM CNS4 on GluN1/2A (A), GluN1/2B (B) and GluN1/2AB(C), GluN1/2C (G) and GluN1/2D (H) receptors. 100 µM glycine was used as co‐agonist. CNS4 reduced glutamate EC_50_ for GluN1/2A [4.42 ± 0.36 µM, *n* = 9 (4 cells) vs. 2.57 ± 0.45 µM, *n* = 5 (4 cells) *p* < .01), and 1/2AB [7.47 ± 1.49, *n* = 5 (4 cells) vs. 1.48 ± 0.33 µM, *n* = 12 (5 cells) *p* < .001], 1/2D [0.34 ± 0.01 µM, *n* = 11 (5 cells) vs. 0.45 ± 0.03 µM, *n* = 11 (5 cells) *p* < .01] receptors. EC_50_ of GluN1/2B [1.97 ± 0.09, *n* = 7 (4 cells) vs. 2.57 ± 0.45 µM, *n* = 5 (3 cells) *p* > .05] and 1/2C [0.47 ± 0.04 µM, *n* = 11 (5 cells) vs. 0.29 ± 0.03 µM, *n* = 11 (5 cells) *p* > .05] receptors in the absence and presence of CNS4 remained unchanged. Values are average ± SEM. Unpaired student's *t* test, *p* < .05. *p < .05, **p < .01, and ***p < .001

Potentiation of NMDAR currents could occur due to various reasons including increased agonist potency, slower desensitization, increased mean open time or channel open probability.^51^ To identify the changes in agonist potency, glutamate dose–response curves were performed in the absence and presence of 30 µM CNS4. Results from these assays revealed that CNS4 significantly increased glutamate potency in GluN1/2A, 1/2AB, and 1/2D receptors (Figure 3A–J). However, CNS4 did not significantly alter GluN1/2B and 1/2C glutamate potency. These findings reveal that at least one of the reasons for CNS4‐induced potentiation of NMDAR currents could be associated with an increase in glutamate potency. Furthermore, these results indicate that minor inhibitions observed with current responses (Figure 2H) were not due to the competitive antagonistic effect of CNS4 at the glutamate‐binding site.

### 3.3 Voltage‐independent and GluN2 subtype‐specific activity of CNS4

**FIGURE 4:**
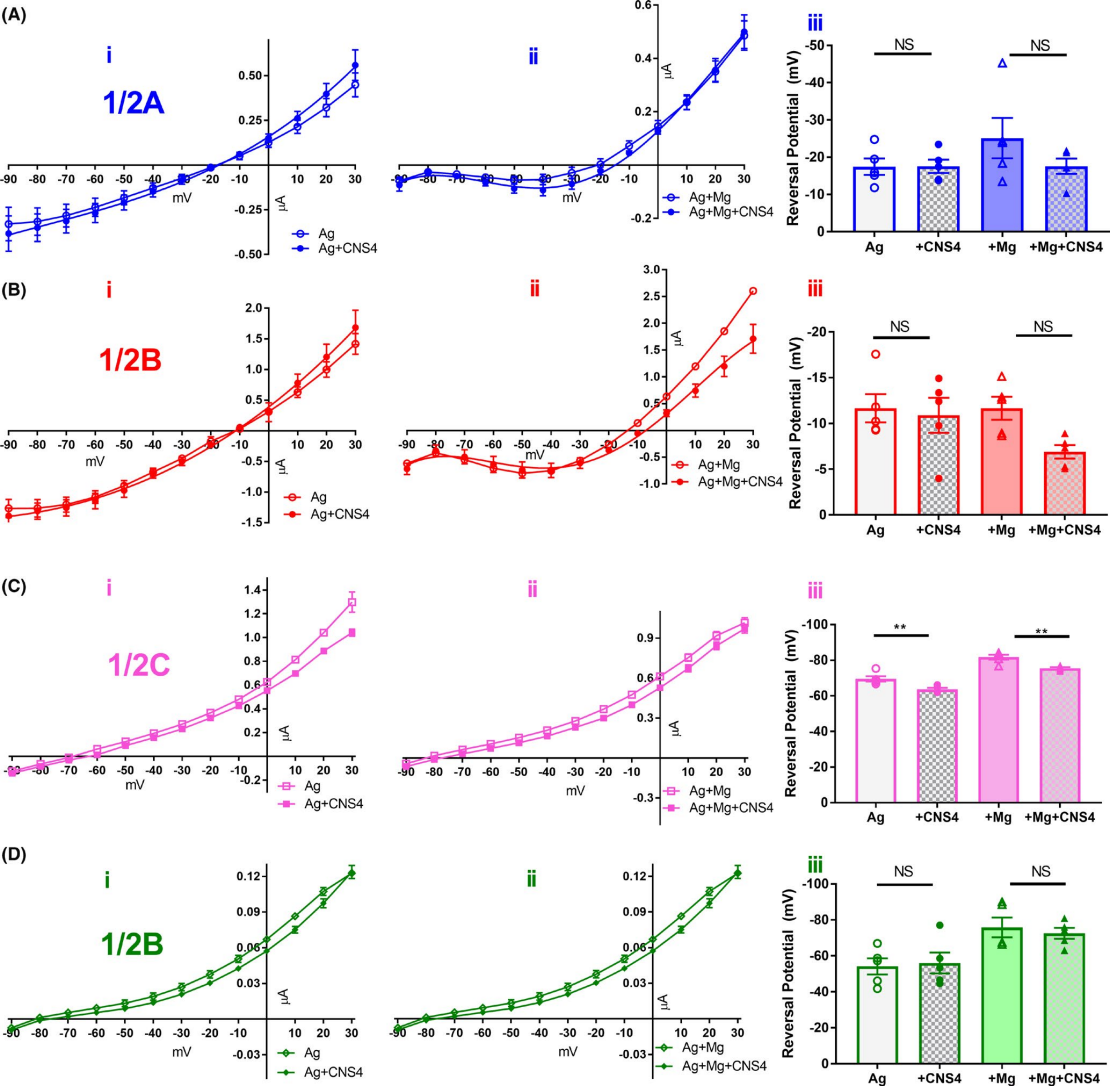
CNS4 activity on NMDA receptor subtypes is voltage‐independent. 100 µM glycine and 100 µM glutamate was used as agonist to activate the receptors. Agonist induced whole cell current–voltage (I–V) relationship was studied in 10 mV intervals ranging from −90 to +30 mv. Reversal potential was obtained (from x‐axis values when *y* = 0.) for each individual recordings and then averaged. In 1/2C receptors, CNS4 altered the reversal potential in the absence (−69.55 ± 1.51 vs. −63.64 ± 0.80 mv, *p* < .01, *n* = 5) and presence (−81.75 ± 1.37 vs. −75.53 ± 0.59 mv, *p* < .01, *n* = 5) of Mg^2+^. A similar reduction was not observed in the absence of Mg^2+^ in 1/2A (−17.42 ± 2.2 mv, *n* = 5 vs. −17.54 ± 1.8 mv, *n* = 5, *p* > .05) or 1/2B (−11.66 ± 1.55 mv, *n* = 5 vs. −10.89 ± 1.9 mv *n* = 5, *p* > .05), 1/2D (−54.2 ± 4.5 mv, *n* = 5 vs. −56.05 ± 5.8 mv, *n* = 5, *p* > .05). In the presence of Mg^2+^, CNS4 did not alter the reversal potential of GluN1/2A (−20.06 ± 2.6 mv, *n* = 4 vs. −17.57 ± 2.0 mv, *n* = 5, *p* > .05) or 1/2B (−11.66 ± 1.25 mv, *n* = 5 vs. −6.9 ± 0.74 mv, *n* = 5, *p* > .05), 1/2D (−75.8 ± 5.51 mv, *n* = 5 vs. −72.58 ± 3.08 mv, *n* = 5, *p* > .05). For each subunit five recordings were made from five different oocytes. Data analysis was blinded and employed. One‐Way ANOVA with Tukey's multiple comparisons test. Current values are obtained from the last 1 s of the 5 s application. NS, not significant

The current–voltage (I–V) relationship studies have been done to determine the voltage‐dependent effect of CNS4. One hundred micrometers glutamate and 100 µM glycine were used as agonists to activate the NMDARs. The agonist‐induced whole‐cell I–V relationship was studied in 10 mV intervals ranging from −90 to +30 mv. CNS4 exhibited no voltage dependence effect on current amplitude in any of the four NMDAR subtypes studied, Figure 4A–D. The highest difference in current amplitude in the presence and absence of 100 µM CNS4 was observed with GluN1/2B at +30 mv. However, this difference was statistically insignificant (1.7 ± 0.26 vs. 2.6 ± 0.36 µA, *p* = .43, *n* = 5, unpaired *t* test), Figure 4Bii. CNS4 plus agonist‐induced currents closely followed the agonist alone current amplitudes throughout the voltage ramp studied. However, in GluN1/2C receptors CNS4 changed the reversal potential of permeant ions (barium and sodium) to less negative compared to the reversal potential of agonist alone experiments. Similarly, in the presence of Mg^2+^, CNS4 reduced the reversal potential in GluN1/2C. However, it had no significant effect on GluN1/2A or 1/2B or 1/2D receptor current reversal potentials, Figure 4A–D. It is noteworthy that GluN1/2A and 1/2B are more sensitive to Mg^2+^ than GluN1/2C and 1/2D receptors,^52^, ^53^ and CNS4 does not alter the reversal potential of permeant ions in 1/2A and 1/2B subunits. I–V experiments were also carried out with 0.3 µM (low) glutamate, and these results are provided as Supporting Information data Figure 4‐1. This set of experiments largely reproduced the results obtained from the 100 µM glutamate assay. However, notably in low glutamate concentration assay, CNS4 changed the reversal potential of permeant ions in both GluN1/2C and 1/2D receptors. This corroborates the potentiation of current by CNS4 for GluN1/2C and 1/2D receptors (Figure 2F,G).

### 3.4 Agonist concentration‐dependent effect of CNS4 in mammalian cells

**FIGURE 5:**
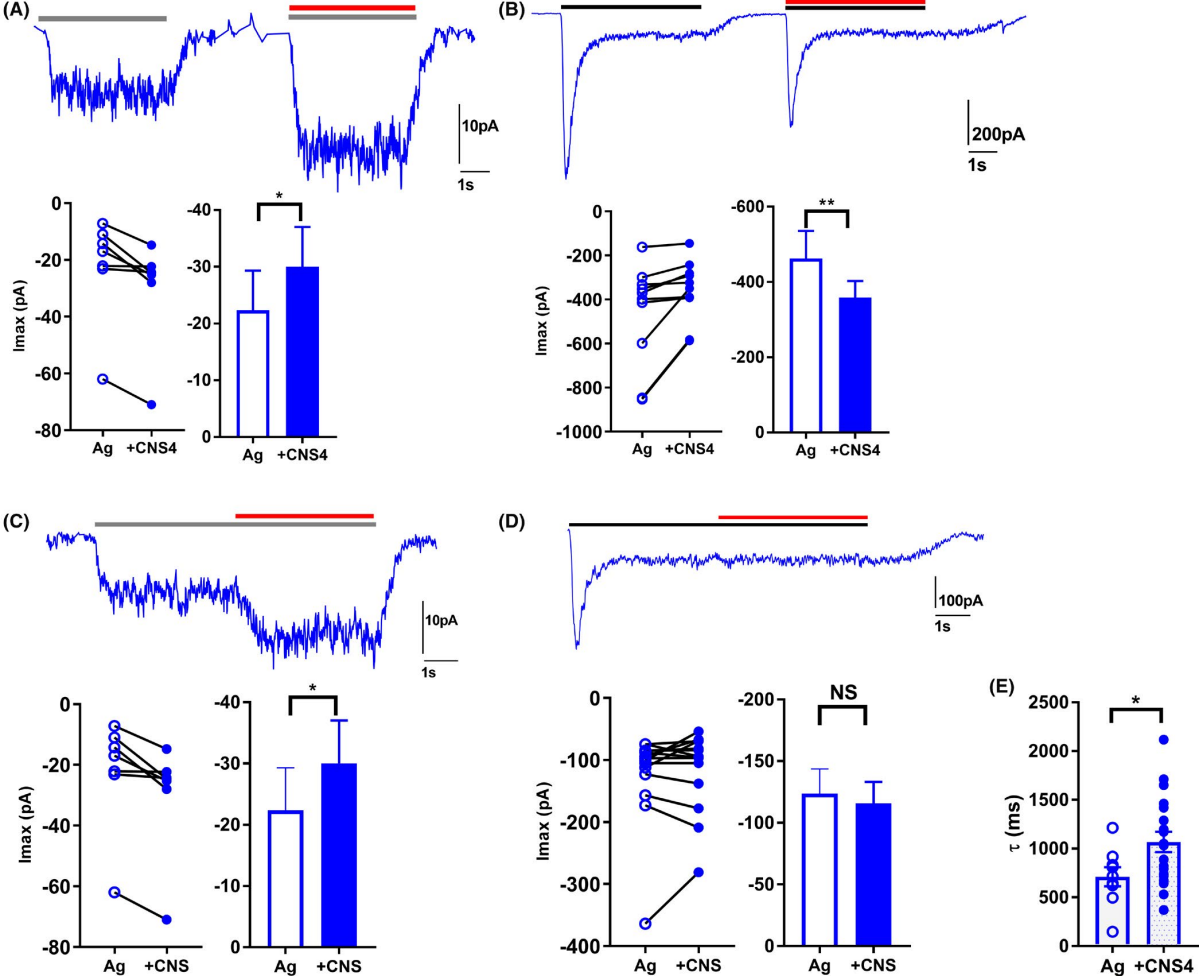
CNS4 modulates GluN1/2A currents in glutamate concentration‐dependent manner. Patch‐clamp electrophysiology assays were performed using HEK293T cells expressing GluN1/2A receptors. Traces represent current responses evoked by 0.3 µM (A, C, gray bar) or 100 µM (B, D, back bar) glutamate and 100 µM glycine as an agonist (Ag). (A, B) 100 µM CNS4 (red) was co‐applied with agonist for 4 s. (C, D) 100 µM CNS4 was applied 4 s after exposure to the respective agonist. Each pair of Ag and +CNS4 application events is shown in dot plots. Histograms show statistical significance. *I*_max_, maximum inducible current by Ag. Steady‐state current values were obtained 4s after Ag application and 4s after +CNS4 application. (A) 0.03 µM Glu, *n* = 8 recordings from four cells. (B) 100 µM Glu, *n* = 10 recordings from three cells. (C) 0.3 µM Glu, *n* = 7 recordings from three cells. (D) 100 µM Glu, *n* = 14 recordings from four cells. Statistics, Wilcoxon matched‐pairs signed rank test. *p < .05; **p < .01. (E) Y‐axis deactivation time constant (τ) in milliseconds. Ag, 100 µM glutamate + 100 µM glycine; +CNS4, 100 µM CNS4 + Ag. CNS4 significantly increased deactivation time constant (Ag, 710.9 ± 98.27 ms, *n* = 9 (three cells) vs. Ag + CNS4, 1067 ± 104.3 ms, *n* = 19 (four cells), *p < .05, unpaired *t* test). NS, not significant

To demonstrate the glutamate concentration‐dependent activity of CNS4 on NMDARs expressed in the mammalian cells, where intracellular scaffolding proteins that are essential for the formation of functional receptors could be different from the ones expressed in xenopus oocytes, we have carried out patch‐clamp electrophysiology assays using HEK293T cells transfected with GluN1/2A receptors. NMDARs were activated by 0.3 or 100 µM glutamate in the presence of 100 µM glycine. CNS4 activity was studied in two different conditions: (1) CNS4 co‐application with agonist (Figure 5A,B), and (2) agonist pre‐application (Figure 5C,D). We hypothesized that these conditions would reveal the effect of CNS4 on the NMDARs that exist in an unbound (apo) state, and receptors that pre‐bound with agonist before binding with CNS4, respectively. As seen in Figure 5, analysis of the maximum inducible currents obtained from agonist and CNS4 co‐application experiments revealed that CNS4 significantly potentiated 0.3 µM glutamate‐induced GluN1/2A currents and significantly inhibited 100 µM glutamate‐induced peak current amplitude (Figure 5A,B). Deactivation time constant tau (τ) was calculated using the exponential weighted fit component of Clampfit 10.7 (pClamp) software. This analysis revealed that CNS4 significantly increased the deactivation time course of GluN1/2A receptors when activated with 100 µM glutamate, Figure 5E (Ag, 710.9 ± 98.27 ms, *n* = 9 vs. Ag + CNS4, 1067 ± 104.3 ms, *n* = 19, *p* < .05, unpaired *t* test). Experiments might require quicker (than ~150 ms ‐used in this study) solution exchange rate or single channel recordings to identify accurate deactivation kinetics. However, comparing two recordings made from the same solution exchange rate could yield the relative difference in deactivation time constant. Thus, these results suggest CNS4 could induce slower dissociation of agonists from the GluN1/2A receptor. Overall, results from these sets of experiments reveal that CNS4 modulates the NMDAR currents based on glutamate concentration.

### 3.5 CNS4 differentially potentiates Ca^2+^ and Na^+^ ion influx in cultured rat brain neurons

**FIGURE 6:**
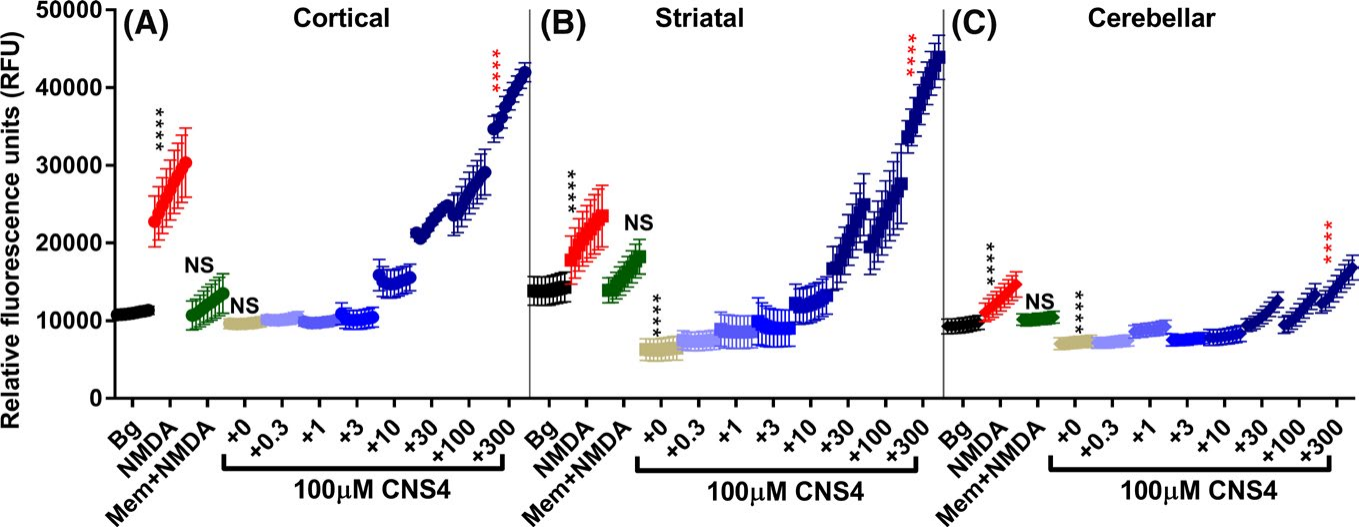
CNS4 potentiates NMDA‐induced Ca^2+^ ion influx in cultured rat brain cortical, striatal and cerebellar neurons. Y‐axis shows relative fluorescence units (rfu). The first two columns (sixteen wells) of the 96 well plate (with DIV14 neurons) served as background (vehicle) control. Each treatment was applied on 8 wells (*n* = 8); there were 10 treatments. In the treatment groups, each data point is an average (and ±SEM bars) rfu value obtained from eight wells; each well was read nine times with a one‐minute interval between each read. Thus there are nine data points for each treatment group. Asterisk colors represent the comparison group. Three different brain regions [cortical (A), striatal (B) and cerebellar (C)] of neuronal origin are labeled. Bg, background; NMDA (red), 300 µM NMDA; Mem + NMDA (green), 100 µM NMDA + 50 µM memantine; The last eight treatments contained 100 µM CNS4 plus different concentrations of NMDA as labeled from +0 to +300 µM (light to dark blue). +0 indicates no NMDA and +300 indicates 300 µM NMDA plus 100 µM CNS4. One‐way ANOVA Tukey's multiple comparisons test was performed to identify the statistical significance between treatment groups. ****p < .0001. NS = not significant

To further study the effect of CNS4 on native NMDARs, we have performed dynamic calcium and sodium imaging assays in cultured rat brain neurons using cell‐permeable Fluo‐8 and CoroNa green AM dyes, respectively. Cortical, striatal, and cerebellar neurons were separately cultured for 14 days in vitro (DIV‐14) before studying the effect of CNS4 as mentioned in the methods section. Results from the Fluo‐8 calcium assay revealed that CNS4 (100 µM) +300 µM NMDA significantly potentiated calcium influx compared to 300 µM NMDA alone in cortex, striatum, and cerebellum (Figure 6A–C). Three‐hundred micrometers NMDA alone significantly increased the Ca^2+^ influx compared to the background control, and addition of 50 µM memantine reversed the NMDA‐induced Ca^2+^ signal back to the control level. These observations confirmed the expression of NMDARs in the cultured neurons and their response to the known pharmacological agents. One hundred micrometers CNS4 in a vehicle (HBSS) with no NMDA produced no significant Ca^2+^ signal in cortical neurons. This indicates CNS4 itself is not activating calcium influx through NMDARs or other endogenous Ca^2+^ ion channels; also not indirectly increasing cytosolic Ca^2+^ levels. CNS4 did not increase Ca^2+^ signal in the presence of 0.3, 1, 3 µM NMDA compared to background (Figure 6A). However, 10 µM or higher concentrations of NMDA + CNS4 significantly increased calcium signals compared to CNS4 alone treated cells. Interestingly, CNS4 plus 300 µM NMDA‐treated cortical cells produced 43% more Ca^2+^ signal compared to plain 300 µM NMDA [mean rfu 26711 vs. 38274, *p* < .0001]. A similar comparison in striatal neurons revealed a 86% of CNS4 induced potentiation (mean rfu 20964 vs. 39001, *p* < .0001). However, CNS4 in vehicle treatment significantly reduced Ca^2+^ signaling in the striatal neurons compared to the background control (mean rfu, 13964 vs. 6317, *p* < .0001). This inhibition was gradually reversed with the increasing concentrations of NMDA. Results from the cerebellar neurons, where relatively less (compared to 1/2A and 1/2B receptors) calcium‐permeable GluN1/2C subunit is predominately expressed,^54^ largely resembled the striatum pattern. Nonetheless, CNS4 plus 300 µM NMDA potentiated Ca^2+^ signal compared to 300 µM NMDA (rfu, 12810 vs. 14423, *p* < .01) in the cerebellar neurons.

**FIGURE 7:**
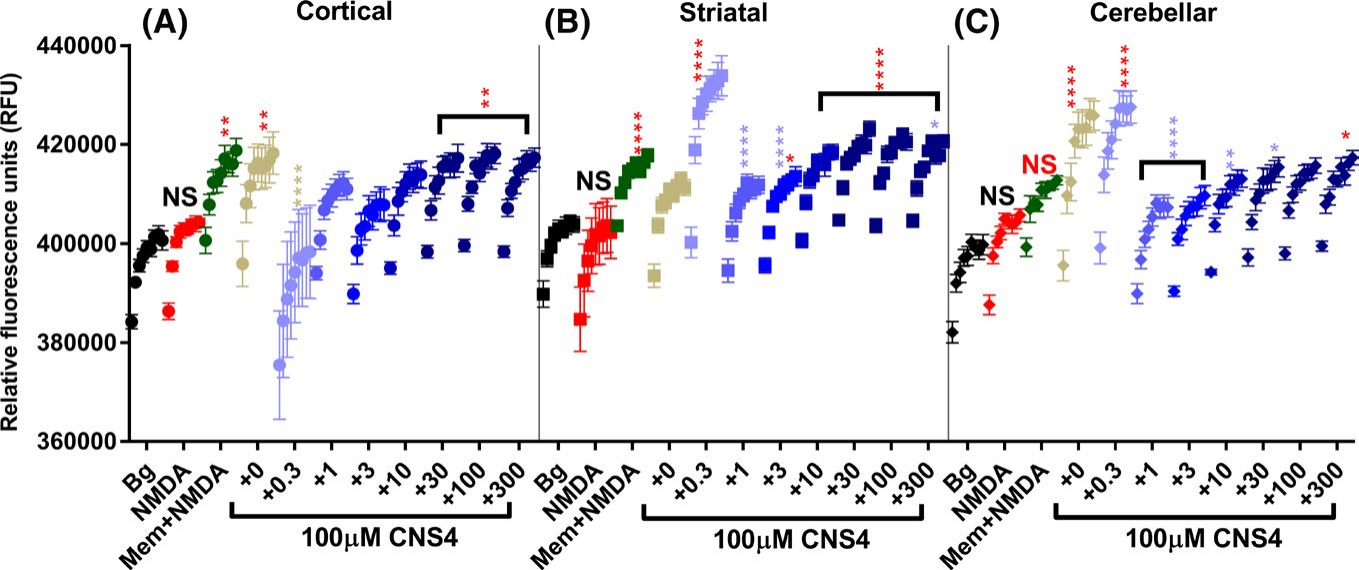
NMDA concentration‐dependent effect of CNS4 on Na+ ions influx in cultured rat brain cortical, striatal and cerebellar neurons. Y‐axis shows the relative fluorescence units (rfu). The first two columns (sixteen wells) of the 96 well plate (with DIV14 neurons) served as background (vehicle) control. Each treatment was applied on 8 wells (*n* = 8), there were 10 treatments. In the treatment groups, each data point is an average (and SEM bars) rfu value obtained from eight wells; each well was read nine times with one min interval between each read. Thus there are nine data points for each treatment group. Asterisk colors represent the comparison group. Three different brain regions [cortical (A), striatal (B) and cerebellar (C)] of neuronal origin are labeled. Bg, background; NMDA (red), 300 µM NMDA; Mem + NMDA (green), 100 µM NMDA +50 µM memantine; 100 µM CNS4 and increasing concentration of NMDA was added, labeled as +0 to +300 µM (light to dark blue). One‐way ANOVA Tukey's multiple comparisons test was performed to identify the statistical significance between treatment groups. *p < .05; **p < .01; ***p < .001; ****p < .0001. NS = not significant

Na^+^ imaging assay carried out using intracellular Na^+^‐binding dye (CoroNa green‐AM) revealed that NMDA did not significantly increase Na^+^ signal in any of the three population of neurons compared to the background control (Figure 7A–C). Remarkably, NMDA plus memantine potentiated the intracellular Na^+^ signal in the cortical neurons compared to NMDA alone (mean rfu, 412868 vs. 400251; *p* < .01). Further CNS4 with no NMDA induced a Na^+^ signal comparable to that of memantine plus NMDA. However, the addition of 0.3 µM of NMDA with CNS4 significantly reduced the sodium signal (mean rfu, 412544 vs. 391591; *p* < .0001). In the presence of 100 µM CNS4, 30–300 µM NMDA produced significantly higher Na^+^ signal than the 300 µm NMDA alone treated cortical neurons (Figure 7A). While a similar pattern was observed with the striatal neurons, there were a few remarkable differences at a sub‐micromolar concentration of NMDA with CNS4. 0.3 µM NMDA plus CNS4 potentiated Na^+^ signal to the highest level (426039rfu) in striatal neurons, as opposed to the inhibition observed in cortical neurons at this concentration. The difference in NMDAR subtype population or other factors might contribute to this difference.

CNS4 alone, and 0.3 µM NMDA plus CNS4, significantly increased Na^+^ signal in the cerebellar neurons (Figure 7C). Notably, 0.3 µM NMDA increased the Na^+^ signal to the highest level in the cerebellar cells (420668rfu). This is consistent with the results obtained from the striatal cells at this NMDA concertation. 3–300 µM NMDA gradually increased the Na^+^ signal in the cerebellar neurons. The memantine‐induced increase in Na^+^ signal does not fit with the expected direction of Na^+^ movement through the NMDA channel. These findings suggest that CNS4‐mediated NMDA concentration‐dependent changes in Na^+^ signals observed might be associated with Na^+^ ion movement through the NMDAR channel.

**FIGURE 8:**
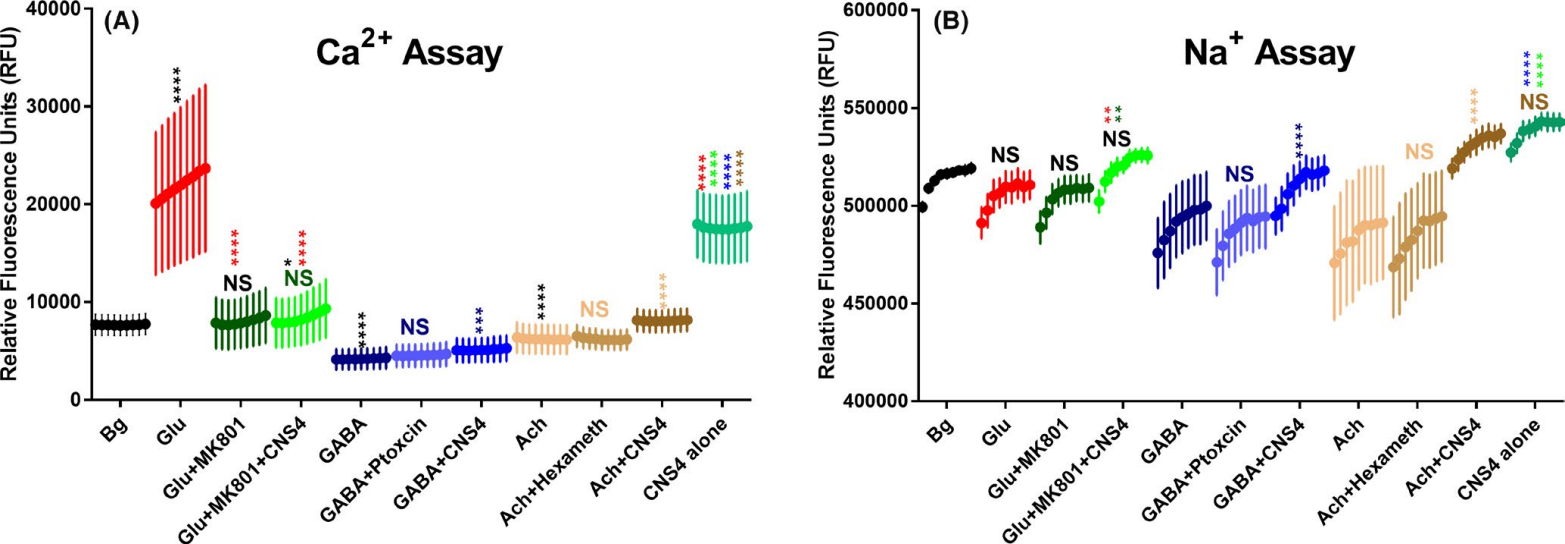
Effect of CNS4 on non‐NMDA glutamate receptors, and GABA and acetylcholine receptors. Ca^2+^ and Na^+^ assay on cultured rat brain cortical neurons following the same procedure as described in the Figures 6 and 7. Y‐axis shows relative fluorescence units (rfu). The first two columns (sixteen wells) of the 96 well plate (with DIV14 neurons) served as background (vehicle) control. Each treatment was applied on 8 wells (*n* = 8); there were 10 treatments. In the treatment groups, each data point is an average (and ±SEM bars) rfu value obtained from eight wells; each well was read nine times with a one‐minute interval between each read. Thus there are nine data points for each treatment group. Asterisk colors represent the comparison group. Bg, background; glu, 100 µM glutamate; MK801, 50 µM; CNS4,100 µM; GABA,100 µM; Ptoxcin, picrotoxcin 50 µM; Ach, acetylcholine 100 µM; Hexameth, hexamethonium, 50 µM. One‐way ANOVA Tukey's multiple comparisons test was performed to identify the statistical significance between treatment groups. *p < .05; **p < .01; ***p < .001 ****p < .0001. NS = not significant.

To study the effect of CNS4 on non‐NMDA glutamate receptors and other major neurotransmitter receptor like GABA and acetylcholine, we have performed an additional set of Ca^2+^ and Na^+^ assays in the cortical neurons. Results from this set of experiments reveal that 100 µM glutamate‐induced Ca^2+^ signal was completely blocked by MK801 [mean rfu 21956 vs. 7997, *p* < .0001], Figure 8A. Furthermore, CNS4 did not significantly increase the Ca^2+^ signals produced by glutamate in the presence of MK801 [mean rfu 7997 vs. 8359, *p* = .7757]. Application of 100 µM GABA significantly reduced baseline Ca^2+^ response [mean rfu 7673 vs. 4196, *p* < .0001], and this GABA mediated effect was unaltered by 50 µM picrotoxin, a GABA receptor antagonist. However, CNS4+GABA made a small but significant increase in Ca^2+^ signal compared to GABA alone [mean rfu 4196 vs. 5124, *p* < .001]. Similar to GABA, acetylcholine also reduced baseline Ca^2+^ signal [mean rfu 7673 vs. 6222, *p* < .0001]. Hexamethonium, a nicotinic acetylcholine receptor antagonist, had no significant effect on acetylcholine‐induced Ca^2+^ signal [mean rfu 6222 vs. 6228, *p* > .9999]. CNS4+acetylcholine significantly increased Ca^2+^ signal compared to acetylcholine alone [mean rfu 6222 vs. 8082, *p* < .0001]. Note: Neither GABA nor predominantly expressing CNS acetylcholine receptors are Ca^2+^ conducting ion channels. Finally, CNS4 alone produced a remarkable increase in Ca^2+^ signal compared to baseline [mean rfu 7673 vs. 17598, *p* < .0001]. This Ca^2+^ signal could come from the ambient glutamate plus CNS4 mediated potentiation of NMDA receptors and activities on other Ca^2+^ channels.

In the Na^+^ assay, glutamate did not significantly alter Na^+^ signal compared to baseline Na^+^ level [mean rfu 514004 vs. 505677, *p* = .4055], Figure 8B. Neither addition of MK801 with glutamate had any effect on the Na^+^ signal. However, the addition of CNS4 with glutamate + MK801 produced a small but significant increase in Na^+^ signal compared to glutamate + MK801 [mean rfu 504283 vs. 519331, *p* = .0024]. Role of CNS4 on GABA and acetylcholine‐induced Na^+^ signals recapitulated the Ca^2+^ assay results. Precisely, CNS4 potentiated both picrotoxin insensitive GABA and hexamethonium insensitive acetylcholine receptor‐mediated Na^+^ signal. Finally, CNS4 alone produced the higher Na^+^ signal (mean rfu 538755) than that was produced in combination with any other agents including glutamate + MK801 (504283) and GABA (491482). Overall, these results indicate that CNS4 directly or indirectly increases intracellular Na^+^ levels through glutamate and non‐glutamate receptors.

## 4 DISCUSSION

Levorotary glutamate is the primary excitatory neurotransmitter in the vertebrate central nervous system.^55^, ^56^ Pulsatile release of glutamate and subsequent changes in glutamate concentration in the synapse are essential for maintaining normal brain physiology.^13^, ^14^, ^15^, ^16^, ^17^, ^18^ Therefore, drugs that modulate NMDARs based on surrounding glutamate concentration could be useful to treat clinical conditions that require enhancing the activity of a subpopulation of receptors that are hypo‐activated either because of insufficient glutamate release or rapid uptake or both. Glutamate concentration biased NMDAR modulators have been recently reported.^57^ In this study, we have identified a chemically distinct small molecule (CNS4) that modulates NMDAR function based on subunit composition and agonist concentration.

Results from the TEVC assay indicated that CNS4 potentiated GluN1/2C and 1/2D receptor currents when activated with a submaximal concentration of glutamate (Figure 2D–H). Interestingly, in the presence of 300 µM glutamate CNS4 had minimal or no activity on any of the five different subtype compositions studied (Figure 2H). Further analysis indicated CNS4 increased glutamate potency in GluN1/2A and 1/2AB receptors and reduced the same in 1/2D receptors (Figure 3). It is noteworthy that CNS4 is not chemically similar to glycine or glutamate. Furthermore, theoretically, the glycine‐binding site in the GluN1 subunit remains identical in all NMDAR subtypes. Therefore, if CNS4 were acting as a glycine site antagonist, it should block all NMDAR subtypes. But this was not observed. These observations indicate that CNS4 differentially modulates glutamate potency, and distinguishes among the closest NMDAR family members, GluN1/2A and 1/2B, and their offspring 1/2AB. I–V experiments revealed when receptors are potentiated the inward currents live longer than the normal activation, thus they might require less negative membrane potential to reverse the current direction. CNS4 might facilitate the inward current of ions in GluN1/2C subunits at both low and high agonist concentrations. However, it does so on GluN1/2D receptors only at low glutamate concentration. Overall, at no voltage step where current responses are significantly different in the presence and absence of CNS4. This revealed that CNS4 activity is voltage‐independent. In the future, physiologically relevant concentrations of permeant ions will be studied to have a better understanding of the changes in reversal potentials observed with GluN1/2C receptors (Figure 4C). Agonist concentration and potentiation pairs observed in patch‐clamp electrophysiology assays do not necessarily match with the equivalent TEVC pair. This could be because of various factors, including the differences in the expression system, state of the receptors when CNS4 molecules approach them, and solution application speed as previously reported.^57^, ^58^, ^59^, ^60^ To further characterize the effect of CNS4 on native NMDARs, we have studied Ca^2+^ and Na^+^ influx through the NMDARs expressed in cultured rat brain cortical, striatal, and cerebellar neurons, after activated with various NMDA concentrations.

CNS4 potentiated 300 µM NMDA induced Ca^2+^ ion influx through native NMDARs expressed in the rat cortical, striatal, and cerebellar neurons (Figure 6A–C). However, CNS4 alone significantly reduced the ion influx in the striatum compared to the background signal. A similar observation was made at the cerebellar cells as well, but not in the cortical cells. This might result from the CNS4‐mediated blockade of NMDARs that are previously activated by the ambient glutamate from the neurobasal media. However, this would lead to a question of why no such reduction was noticed in the cortical neurons? A plausible explanation could be that the spatiotemporal expression of NMDAR subtypes in the cortex, striatum, and cerebellum may play a role in this difference.^61^ GluN1/2AB receptors are found to be predominantly expressed in the cortical neurons. In the striatum, in addition to GluN1/2A and GluN1/2B receptors, GluN1/2D receptors are also expressed.^62^ It is possible that ambient glutamate might preferentially activate GluN1/2D receptors since this subtype of NMDARs has about three‐ to six‐fold higher affinity for glutamate compared to GluN1/2A or 1/2B receptors.^63^ This notion would also be consistent with the reduction in Ca^2+^ signals observed with CNS4 alone in the cerebellar neurons where GluN1/2C receptors are predominantly expressed. GluN1/2C receptors also have higher glutamate affinity compared to the canonical NMDAR subunits. Thus, CNS4 alone could have blocked the ambient glutamate‐induced activation of GluN1/2C & 1/2D receptors expressed in the cerebellar and striatal neurons.

Interestingly, memantine significantly increased intracellular Na^+^ signal in cortical and striatal neurons, in contrast to a channel blockade‐mediated reduction (Figure 7A,B). This could have happened due to the activity of various sodium channels expressed in the neurons (no Na^+^ channel blocker was used in the assay). Therefore, this observation might indicate either Na^+^ influx through the NMDA channel was not blocked by memantine or memantine induced NMDAR blockade might indirectly increase Na^+^ influx into the neurons to maintain the intracellular electrolyte homeostasis, as previously reported.^64^, ^65^ Furthermore, CNS4 alone also increased the Na^+^ signal in neurons from cortical and cerebellar neurons compared to 300 µM NMDA alone (Figure 7A,C). However, the addition of as little as 0.3 µM NMDA (note: NMDA is a weak agonist of the NMDAR with about three‐fold less potency than glutamate^66^ created turbulence in the Na^+^ signal. For example, in the cortical neurons addition of 0.3 µM NMDA with CNS4 produced the lowest level of Na^+^ signal which is opposite to the highest level of Na^+^ signal observed with CNS4 alone. In contrast, 0.3 µM NMDA produced the highest level of Na^+^ signal observed both in striatal and cerebellar neurons. These findings suggest that the changes in sodium signals observed with memantine or CNS4 alone could be associated with NMDAR channel activity and not completely due to non‐NMDA channel activity. Overall, observations made from the Na assay suggest that CNS4 evokes a distinct agonist concentration‐dependent Na^+^ influx through the native NMDARs. The results obtained from the Ca^2+^ and Na^+^ assay with focus non‐NMDA glutamate receptors and GABA and acetylcholine receptors indicate that CNS4 might have direct or indirect activities on non‐NMDA glutamate and other receptors.

### 4.1 Limitations & future directions

Co‐crystallization of CNS4 with NMDAR subunits could not be performed in this study. Previous studies reported that agonist‐mediated cascades of conformational changes occurring in the extracellular domains of NMDAR determine distinct biophysical properties, downstream signaling mechanisms, and pathogenesis of cognitive dysfunction.^5^, ^67^, ^68^, ^69^ Furthermore, NMDARs undergo at least two glutamate concentration‐dependent distinct desensitization states as previously reported.^70^, ^71^ Among these two, one results from the weakening of glutamate affinity immediately after channel opening and the other form of desensitization occurs when channels enter into a long‐lived non‐conducting state. Both glutamate‐ and glycine‐binding and dissociation rate directly contribute to these different desensitized states.^70^ We hypothesize that CNS4 might bind with the NMDAR subtypes at more than one site as they are generated by different concentrations of agonists. Future investigations on the binding sites will provide a better understanding of the molecular pharmacology of this compound. Overall, CNS4 and its future analogs will serve as a chemical tool to study the biology of NMDARs, and as lead candidates to develop clinically useful antipsychotic drugs with fewer on‐target adverse effects.

## DISCLOSURE

The authors declare no competing financial interest.

## AUTHORS CONTRIBUTION

BMC contributed to conceptualization, project administration, and manuscript writing. BMC and BGK contributed to funding acquisition and progress reports. BMC, LCK, BM, DNB, BNV, and TVJ contributed to experiments. BMC, LCK, DNB, BNV, and RR contributed to data analysis. DM contributed to chemical synthesis.

## NOMENCLATURE OF TARGETS AND LIGANDS

Key protein targets and ligands in this article are hyperlinked to corresponding entries in http://www.guidetopharmacology.org, the common portal for data from the IUPHAR/BPS Guide to PHARMACOLOGY,^72^ and are permanently archived in the Concise Guide to PHARMACOLOGY 2019/20.^73^

## Supporting information

Fig S1‐S4

## Data Availability

The datasets generared during the current study are available from the corresponding author on reasonable request.
