# Supplementary material for: A glutamate concentration‐biased allosteric modulator potentiates NMDA‐induced ion influx in neurons
Source: Pharmacol Res Perspect. 2021 Sep 3;9(5):e00859. doi: 10.1002/prp2.859 (PMC8413904; doi:10.1002/prp2.859)
Supplement: Supplementary file 1 — Fig S1‐S4 [file PRP2-9-e00859-s001.pdf]

**Supplemental data figure 1-1:**

Synthesis of 4-fluoro-N-(2-(pyridin-3-yl)piperidine-1-carbonothioyl)benzamide (CNS4): Anabasine, piperidinylpyridine alkaloid, based new thiourea was synthesized in two steps synthesized using thiocarbamoylation reaction. The starting 4-fluorobenzoyl isothiocyanate was synthesized *in situ* by heating 4-fluorobenzoyl chloride **1** with potassium thiocyanate in acetone. Further reaction of fluorobenzoyl isothiocyanate **2** with anabasine **3** in THF at room temperature yielded 4-fluoro-N-(2-(pyridin-3-yl)piperidine-1-carbonothioyl)benzamide (**CNS4**). The synthesized compound was confirmed by $^1$H-NMR & LCMS analysis and HPLC purity >99%.

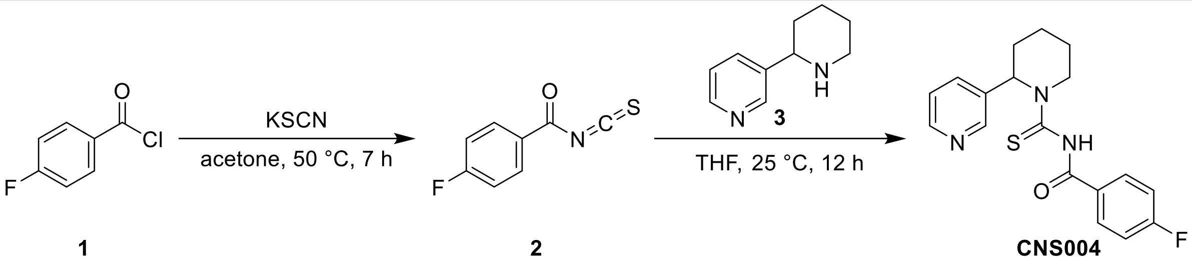


**Experimental:**

**4-Fluorobenzoyl isothiocyanate (2):** To a stirred solution of 4-fluoro benzoyl chloride **1** (2 g, 12.613 mmol) in dry acetone (15 mL) at room temperature under atmosphere was added potassium thiocyante (1.47 g, 15.136 mmol) at once. The reaction mixture was heated at 50 °C for 5 hours. After completion, the reaction mixture was filtered through a celite bed washed with acetone to remove inorganics. The filtrate was concentrated under *vacuo*. The obtained crude mixture was dissolved in a mixture of DCM/hexane (20 mL, 1:1 ratio) and passed through a pad of silica gel (230-400 mesh). The solvent was then evaporated under *vacuo* to get compound **2** gummy as a red colour solid, as such taken for the next step without further purification (Yield: 1.3 g).

**4-Fluoro-N-(2-(pyridin-3-yl)piperidine-1-carbonothioyl)benzamide (CNS4):** To a stirred solution of anabasine **3** (0.1 g, 0.616 mmol) in dry THF (5 mL) at room temperature under nitrogen atmosphere was added compound **2** (0.11 g, 0.616 mmol) at once. The reaction mixture was stirred at room temperature for 16 hours. After completion, the reaction mixture was concentrated under *vacuo*. The crude mixture obtained was further purified by flash column chromatography on silica gel, 230-400 mesh using 20-25% of ethyl acetate in petroleum ether as an eluent to get **CNS4** as a pale-yellow solid (Yield: 0.13 g; 39% over two steps). $^1$H-NMR (400 MHz, DMSO-$d_6$): δ 10.99 (br s, 1H), 8.72 (br s, 1H), 8.52 (d, J = 4.52 Hz, 1H), 8.08 (br s, 2H), 7.92 (br s, 1H), 7.46 (dd, J = 4.80, 7.92 Hz, 1H), 7.39-7.35 (m, 2H), 6.82 (br s, 1H), 4.02 (br s, 1H), 3.03 (dt, J = 5.16,

Hz, 1H), 2.63-2.60 (m, 1H), 1.98 (br s, 1H), 1.65-1.62 (m, 3H), 1.42-1.40 (m, 1H); **LC_MS:** Calc. for $C_{18}H_{18}FN_3OS$, 343.42; Obs. 344.2 $[M+H]^+$.

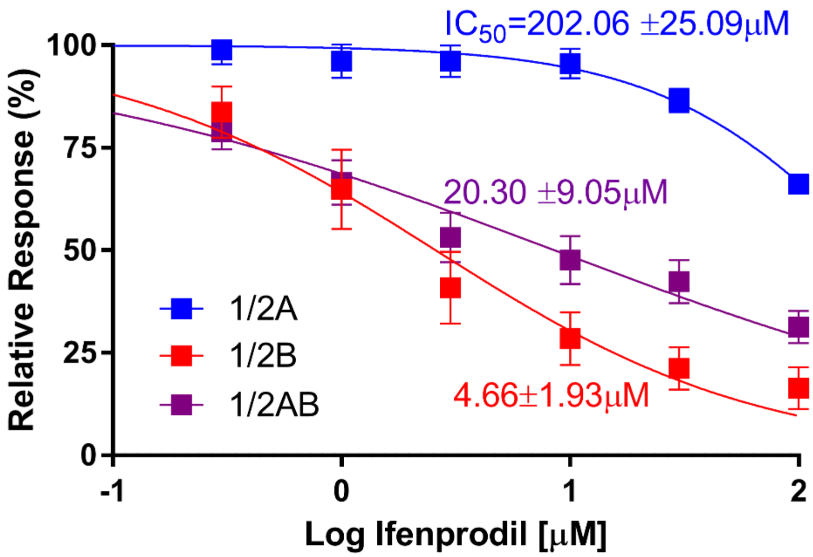


**Supplementary data Figure 2-1:**

**Ifenprodil inhibits GluN1/2AB receptors with an IC50 that is intermediate to GluN1/2A and 1/2B receptors.** Ifenprodil dose-response curve was performed in the presence of 100µM glutamate and 100uM glycine concentration. GluN1/2A (n=5), 1/2B (n=6) & 1/2AB (n=8). IC50 values obtained by a non-linear fit are provided in the figure.

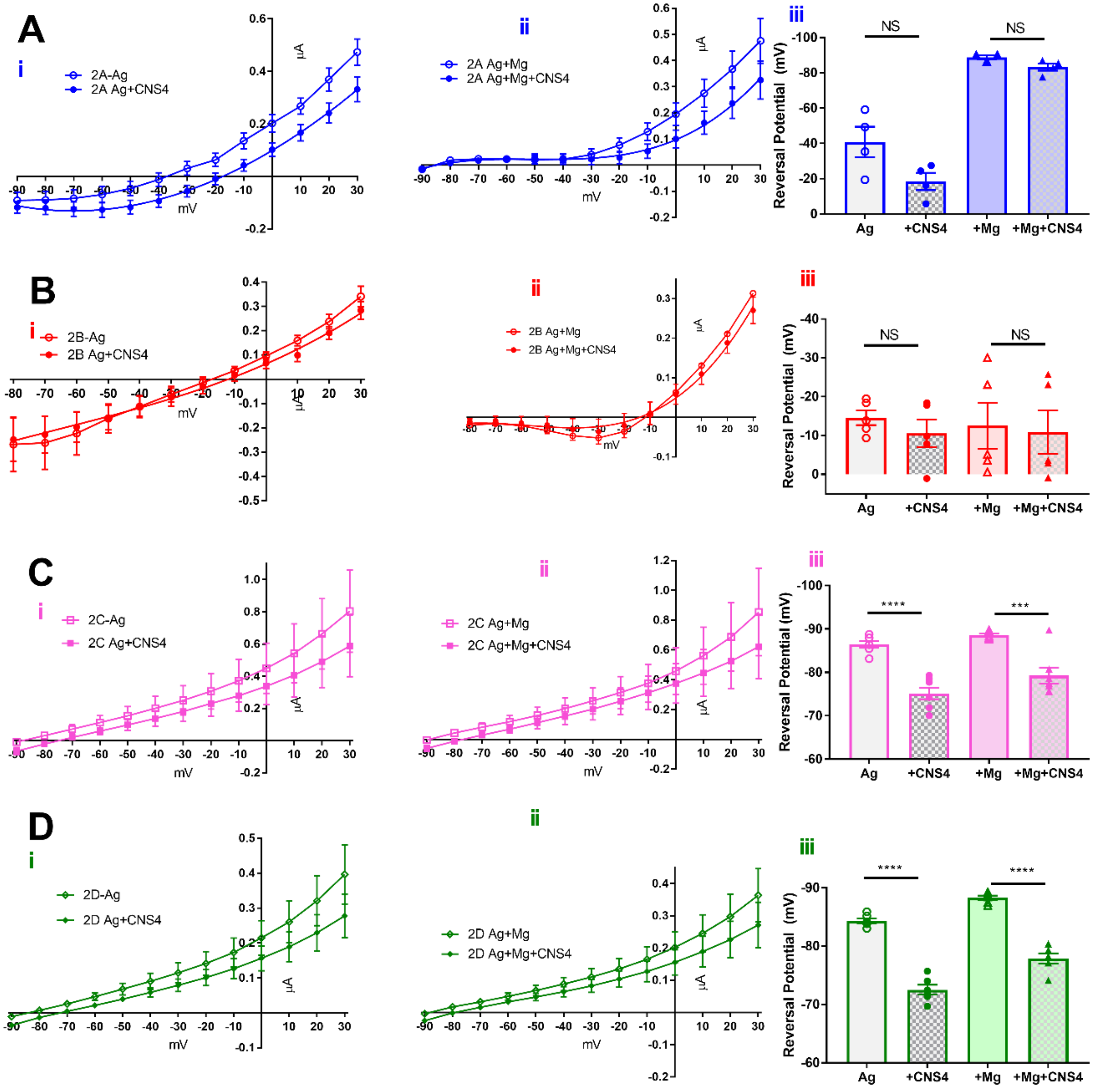


**Supplementary data Figure 4-1.** Voltage independent activity of CNS4. 100uM glycine and 0.3uM glutamate were used as agonists to activate the receptors. Agonist-induced whole-cell current-voltage (I-V) relationship was studied in 10mV intervals ranging from -90mv to +30mv. In GluN1/2C receptors, CNS4 altered 0.3uM glutamate (A,B,C,&D) induced inward current reversal potential in the absence (-86.44± 0.74 vs -75.15±1.33 mv, $p<0.0001$, $n=7$) and presence (-88.59± 0.30 vs -79.25±1.87mv, $p<0.001$, $n=7$) of Mg2+. A similar reduction was observed with GluN1/2D receptors in the absence (-84.35± 0.42mv vs -72.52±0.85mv, $p<0.0001$, $n=6$) and presence (-88.29± 0.35mv vs -77.88±0.87mv, $p<0.0001$, $n=6$) of Mg2+. Each subunit recordings were made from 5-7 oocytes. Data analysis was blinded and employed One-Way ANOVA with Tukey's

multiple comparisons test. Current values are obtained from the last one second of the 5-second application. NS, not significant.
